# Microridges are apical epithelial projections formed of F-actin networks that organize the glycan layer

**DOI:** 10.1038/s41598-019-48400-0

**Published:** 2019-08-21

**Authors:** Clyde Savio Pinto, Ameya Khandekar, Rajasekaran Bhavna, Petra Kiesel, Gaia Pigino, Mahendra Sonawane

**Affiliations:** 1 Department of Biological Sciences, Tata Institute of Fundamental Research, Colaba, Mumbai, India; 2 Max Planck Institute of Molecular Cell Biology and Genetics, Dresden, Germany

**Keywords:** Actin, Differentiation

## Abstract

Apical projections are integral functional units of epithelial cells. Microvilli and stereocilia are cylindrical apical projections that are formed of bundled actin. Microridges on the other hand, extend laterally, forming labyrinthine patterns on surfaces of various kinds of squamous epithelial cells. So far, the structural organization and functions of microridges have remained elusive. We have analyzed microridges on zebrafish epidermal cells using confocal and electron microscopy methods including electron tomography, to show that microridges are formed of F-actin networks and require the function of the Arp2/3 complex for their maintenance. During development, microridges begin as F-actin punctae showing signatures of branching and requiring an active Arp2/3 complex. Using inhibitors of actin polymerization and the Arp2/3 complex, we show that microridges organize the surface glycan layer. Our analyses have unraveled the F-actin organization supporting the most abundant and evolutionarily conserved apical projection, which functions in glycan organization.

## Introduction

Epithelial tissues cover the outer surface of metazoans as well as line the lumen and outer surfaces of organs. Over the course of evolution, epithelia have undergone diversification and specialization allowing them to adapt to various conditions and perform not only the barrier function but also additional functions such as absorption, secretion and mechanosensation^1,2^. These functions require special adaptions of their apical surfaces^3,4^, which include diverse apical membrane protrusions. Additionally, the apical surface is kept hydrated and protected from pathogens and physical injury by the overlying glycan layer^3,5,6^. Thus, the apical zone of epithelial tissues is comprised of three components, namely, the apical plasma membrane with its projections, the cytoskeleton supporting these projections and the outer glycan layer^3^.

In vertebrates, epithelial cells exhibit membrane protrusions of various shapes and sizes. Columnar epithelia possess actin-based cylindrical microvilli or their derivatives such as stereocilia^3,7,8^. On the contrary, the apical domain of several of the non-cornified squamous epithelia display long laterally arranged protrusions called microplicae or microridges^3,9^. While previous studies have significantly enhanced our understanding of the actin organization in microvilli^7,10^, how actin is organized to build microridges remains controversial.

Mature microridges are relatively stable wall-like protrusions, arranged in a labyrinthine fashion^11–16^. They are shown to be present on several non-cornified epithelia in various species across vertebrate phyla, making them one of the more widespread actin protrusions^9,11,17–26^. Microridges are thought to have important roles in mucus retention, abrasion resistance and increasing the tensile strength of the apical domain^9,14,27,28^. They have also been proposed to function as a rapidly deployable store of F-actin and membrane during wound healing^9,28^.

The nature of the protrusion is determined by the organization and dynamics of F-actin within it. Filaments in actin protrusions take on one of two conformations - either as a branched network of filaments, as seen in large parts of the lamellipodium^29^, or as parallel bundles forming the core of projections like microvilli and stereocilia^8,30^. Although the importance of the Arp2/3 complex, an important regulator of branched actin networks^31^, in the maintenance of microridges in zebrafish epidermal cells is known^13^, it is not clear how actin within them is organized. While actin within microridges of guppy epidermal cells is reported as bundled^11^, microridges on carp oral mucosal cells, cat epithelial cells, and guppy cells in culture are shown to contain a network of actin^11,20,32^. Furthermore, based on scanning ion-conductance microscopy in A6 cells, it has been proposed that microvilli can merge to form ridges^33^, suggesting that microridges form from microvilli containing F-actin bundled cores.

The zebrafish periderm offers a genetically and microscopically tractable model to study the formation, structure and function of microridges^13^. It has been recently shown that the cell polarity regulator atypical Protein Kinase C (aPKC) plays a role in restricting the elongation of microridges by controlling levels of Lethal giant larvae (Lgl) and nonmuscle Myosin II at the apical domain of peridermal cells^34^. However, the ultrastructure and function of these protrusions have not been characterized well. Here, we have performed molecular as well as structural analysis of microridges in the developing zebrafish epidermis and probed for their functional importance in the context of the glycan layer. We show that the molecular composition of microridges is similar to lamellipodia and that the Arp2/3 complex function is essential for their formation and maintenance. Electron tomography (ET) followed by segmentation analysis reveals that the actin is organized in the form of a network. We further show that microridges are formed from small actin punctae that contain the Arp2/3 complex. Lastly, our analyses also indicate that microridges are important for the organization of the glycan layer.

## Results

### Ultrastructural characterization of the apical zone of peridermal cells

The ultrastructural characterization of the entire apical zone including the microridge and its surroundings has not been carried out so far in the developing zebrafish periderm. We looked at the organization of the peridermal cell in transmission electron micrographs (TEM) as well as in scanning electron micrographs (SEM) at 48 hpf — a time point at which embryos begin to hatch and exhibit well formed microridges^34^.

**Figure 1:**
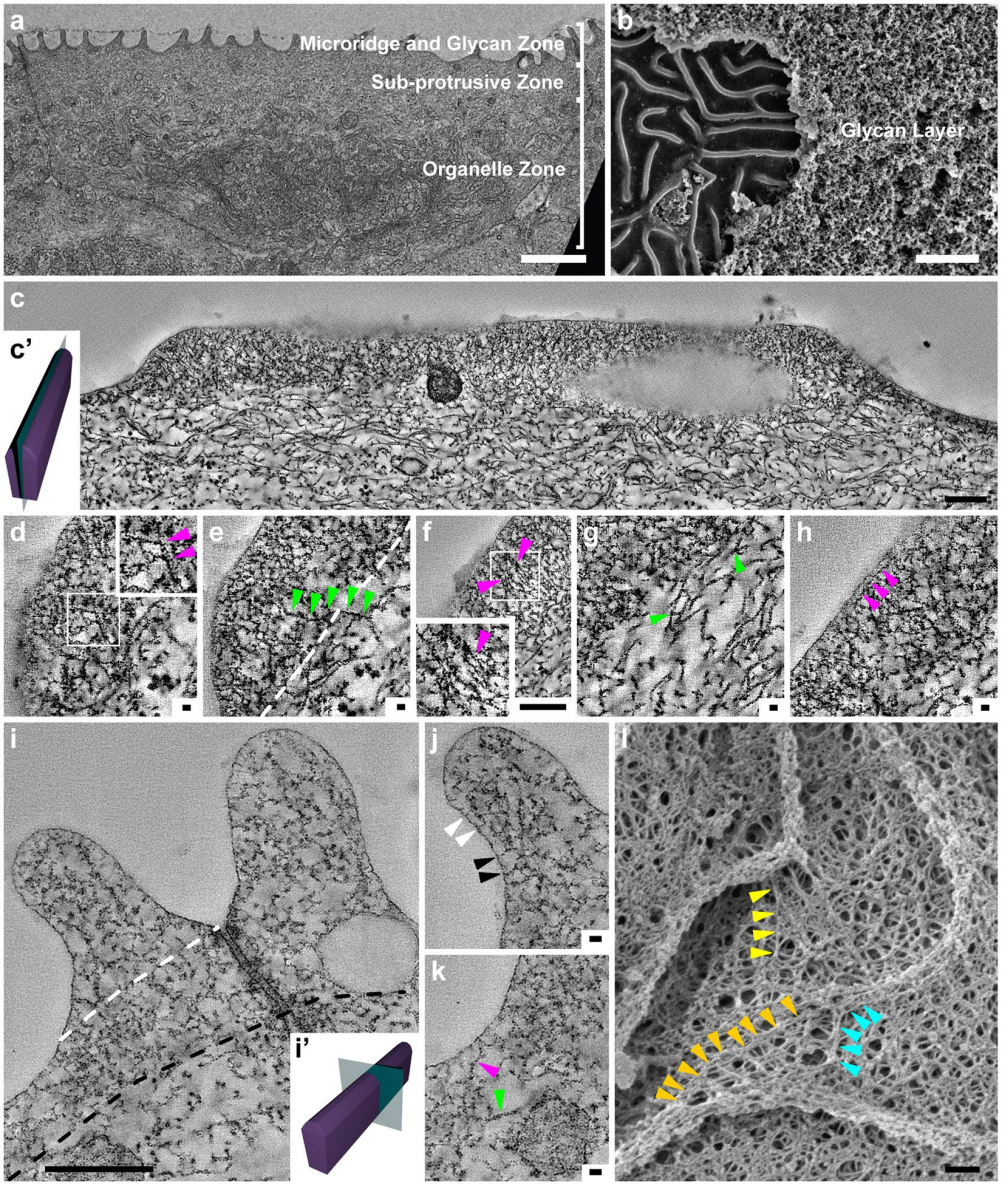
EM tomography of untreated samples and SEM on detergent extracted samples reveals the ultrastructural organization of the actin microridge, sub-protrusive zone and cortex. TEM through a head peridermal cell revealed that peridermal cells comprise of a microridge/glycan zone, sub-protrusive zone and organelle zone (**a**). SEM of an Alcian blue and lysine fixed head periderm sample (**b**) reveals a thick glycan layer, which is chipped off on the left revealing the underlying microridges. Tomograms of Gluteraldehyde + OsO_4_ fixed samples through the length of the microridge (**c**) and perpendicular to the cell-boundary microridge (**i**). Illustrations of the sectioning angle (c’, i’) with the microridge (in purple) and the sectioning plane (in green) for c-h and for i-k, respectively. Zoomed in areas of tomograms in c and i are shown in (**d**–**h**) and (**j**,**k**), respectively. Each image in c-h and i-k represents a 0.7 and 0.55 nm thick slice of the tomogram in the Z axis respectively. SEM of a detergent extracted sample (**l**). Magenta and green arrowheads indicate actin and keratin filaments, respectively. In the inset in (**d**), the arrowheads point to a filament with branch points. In (**e**) the arrowheads point to a keratin filament entering the microridge from the sub-protrusive zone whereas the white dashed line demarcates the boundary between the microridge above from the sub-protrusive zone below. Small filaments that appear to be parallel to each other (**f**) are shown by the arrowheads and inset in f. The arrowheads in (**g**) point to branch points in keratin filaments in the sub-protrusive zone. In (**h**) the arrowheads point to a filament that is parallel to the plasma membrane. The cytoskeleton from the top of the microridge to its base (indicated by white dashed line in ‘**i**’) and the sub-protrusive zone till the base of the tight junction (indicated by the black dashed line) is denser and made up largely of actin. In (**j**) smaller actin membrane crosslinks (white arrowhead) and longer actin membrane crosslinks (black arrowhead) are shown. The cortex between ridges (**k**) is made up largely of F-actin, which are thinner filaments (magenta arrowhead), distinctly different from the thicker keratin filament below (green arrowhead). Coloured arrowheads in (**l**) indicate filaments or filament bundles extending from the cortex into the microridge. All TEM images are taken on top of the head, while the SEM is from on top of the yolk. All samples were fixed at 48 hpf. The scale bars in a,b are 2 µm, in c,f,i,l are equal to 200 nm and those in d,e,g,h,j,k are 20 nm.

Routine TEM analysis using glutaraldehyde (GA) and osmium tetroxide (OsO_4_) fixation revealed three distinct zones in the peridermal cell - the protrusive zone consisting of microridges, the sub-protrusive zone comprised of actin and keratin filaments and a basal region containing various organelles (Fig. 1a). Microridges had a mean height of 315 ± 82 nm (n = 245, N = 3; Fig. S1b), as measured from TEM. Using SEM (Fig. S1a), we found their mean width to be 162 ± 36 nm (n = 645, N = 3; Fig. S1b). To observe the organization of the glycan layer, we fixed samples in the presence of alcian blue and lysine, which has been shown to preserve the glycan layer better^35–37^. SEM of heads of animals fixed in such a way showed that the peridermal surface was covered by a thick glycan layer, which completely obscures the underlying microridges on the head epidermis (Fig. 1b). TEM analysis of such a sample revealed glycan enrichment around the microridges (Fig. S1c).

**Figure 2:**
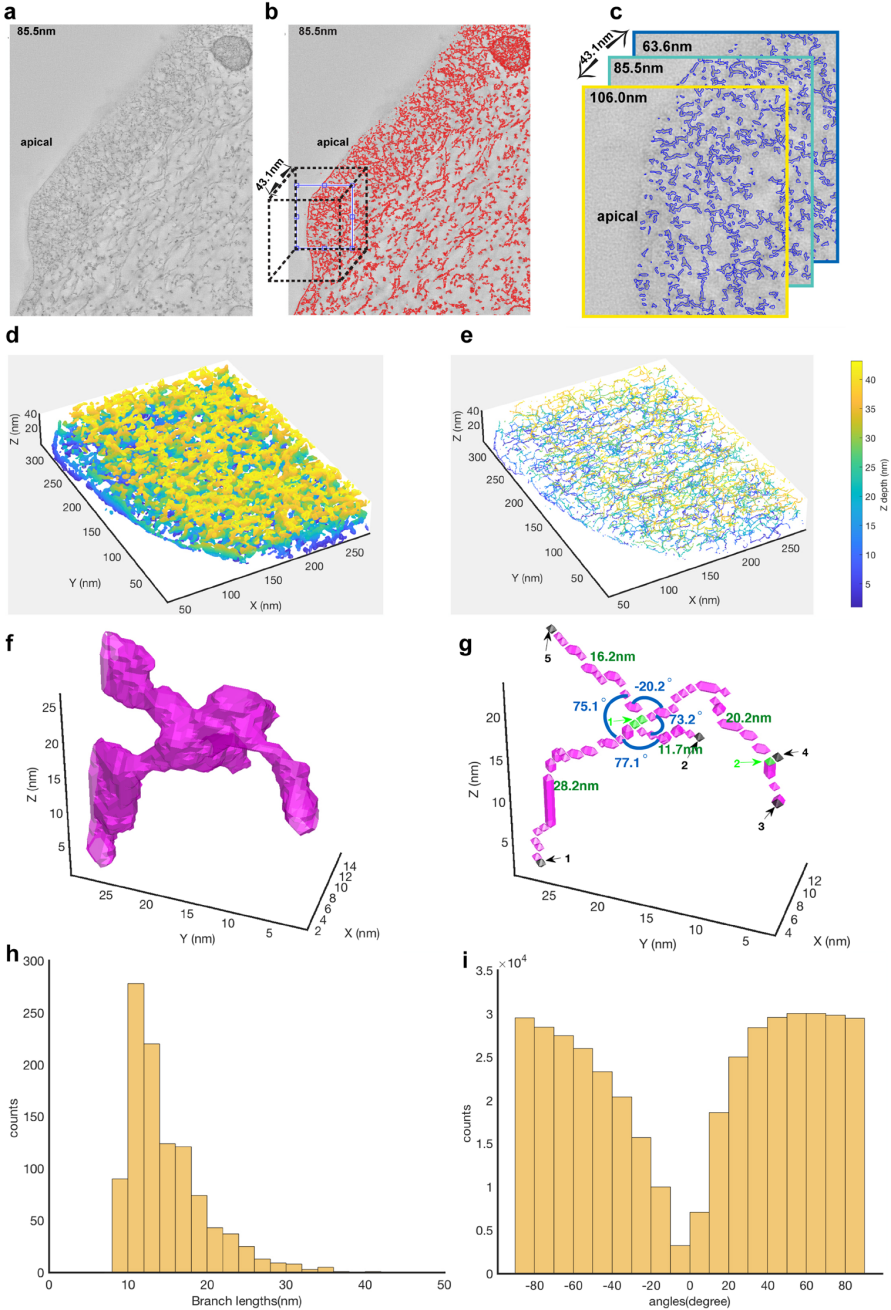
Segmentation of an electron tomogram reveals the arrangement of actin as a network within the microridge. An EM image section tomogram was taken with 2090 × 1740 × 249 voxels at an equal spacing of 0.707 nm in each direction, here shown at a depth of 85.5 nm (**a**). Higher eigenvalue 2D matrix outlines the actin structures within the microridges shown in (**b**). A cubic section of 283.5 × 318.8 × 43.1 nm^3^ (dotted cube in b) is cropped for illustration of the segmentation analysis. Image binarization (in blue) outlines the actin structures (**c**), shown at 3 different depths. 3D reconstruction of actin (**d**) and corresponding 3D skeleton image (**e**) reveal the arrangement of actin meshwork. Depth is indicated by colorbar. An example of actin filament rendering in magenta (**f**) and corresponding skeleton image (**g**) from an interconnected microridge. Branch points (green) and endpoints (black) are highlighted on the 3D skeleton images showing branch lengths (in nm) and angle between neighboring branches. All the branch points and endpoints are numbered and indicated by arrow marks. Quantitative analysis from image segmentation yielded branch lengths (**h**) to be within the range of 10–20 nm. Angles between neighboring microridge branches emanating from a common branch point followed a wide trend ranging from −90° to +90° but most falling in the range between ±60° to ±90° (**i**). The EM tomogram analyzed here (**a**–**c**) was a part of the tomogram shown in Fig. 1c–h and Video 1.

Since conventional TEM did not reveal the F-actin organization within the microridge due to the dense packing of actin (Fig. S1d), we used Electron Tomography (ET) to unravel the structural details of actin organization (Fig. 1c–k). We produced tomograms (Videos 1–3), with different orientations of microridges. Samples fixed using osmium and GA (Fig. 1c–k; Videos 1; 2) showed that filaments within a microridge were largely arranged at different angles to each other (Fig. 1c–j), suggestive of a network of actin along with a few parallel filaments (Fig. 1f). Aside from a few filaments parallel to the microridge membrane (Fig. 1h), we observed a number of actin-membrane crosslinks at the interphase between the membrane and the actin network (Fig. 1j). Interestingly, there were many instances in which vesicles were found in the microridge, particularly at its base, indicating that it is not an isolated system like a cilium (Figs 1i; S1e). In order to obtain a more detailed view of the actin organization, we used an algorithm (see methods) to segment filaments in a tomogram of microridges (Fig. 2). This allowed reconstruction of the filaments within the microridge as shown, within a small region of the tomogram (Fig. 2b–f, Video 4). These corroborated the fact that filaments are organized in a network like fashion (Fig. 2e; Video 4). We were also able to observe branching of filaments (Figs 1d; 2f,g). Similar tomography and segmentation analysis was done on samples fixed using GA, tannic acid and stained en bloc using uranyl acetate, avoiding OsO_4_ — to test whether the fixation influences the actin organization (Fig. S2). This analysis corroborated the fact that actin within the microridge is organized as a network (Video 3; Fig. S2e). The segmentation analyses of this tomogram further revealed that, irrespective of the fixative used, filament branch lengths were found to be within the range of 10 to 20 nm (Figs 2g,h; S2g,h). The angles between neighboring filaments followed a wide distribution with the majority of angles within the range of ±60° to ±90° (Figs 2i; S2i).

To validate the performance of the segmentation algorithm, we generated artificial images (Fig. S3, see methods for details) with similar characteristics as that of EM microridge filaments and segmented these using the parameters used in Fig. 2. This analysis revealed that our segmentation method has a sensitivity of 79.1% as compared to manually-segmented ground-truth.

Our ET analysis further revealed that the sub-protrusive zone contained cortical actin and a network of thick keratin filaments with discernible branching points (Fig. 1c,g,i,k). Some keratin filaments —discernable by their thickness as compared to actin (Fig. 1k) — clearly entered the main body of the microridge, indicating that keratin and actin filaments arising from the cortex support its architecture (Fig. 1e). To confirm such a contribution from the cortex, we used detergent extraction to remove apical membranes of peridermal cells and performed SEM. In these preparations, actin filaments in the cortex were clearly visible (Fig. 1l). These filaments were arranged in a web-like pattern. Interestingly, we found that actin filaments and filament bundles extended a considerable distance within the cortex and into the microridge (Fig. 1l). Further corroborating with these ET and SEM data, we observed the localization of keratin - using the AE1/AE3 antibody - to the apical cortex and occasionally in the microridge (Fig. S4a). We did not observe any direct association of microtubules with the microridge either by ET or immunostaining using an α-tubulin antibody (Fig. S4b).

To ascertain that GA fixation preserves bundled actin in zebrafish, we inspected microvilli from a 6-day-old zebrafish larval intestine. Although fixed using a modified version of the GA fixation protocol^38^ than used here, ET of this sample showed clear bundles of actin within microvilli, along with the presence of a dense zone at their tips, which is the tip complex (Video 5)^7^. This analysis suggests that the absence of bundled actin in microridges is unlikely to be a consequence of improper fixation.

To conclude, the apical zone of zebrafish peridermal cells consists of a glycan layer, an apical domain consisting of short projections called microridges and the cytoskeleton that supports them. Within the microridge, F-actin is organized in the form of a network, supported by filaments arising from the sub-protrusive zone or terminal web and cortex. The interior of the microridge space is accessible to vesicles indicating that the microridge is not an isolated system.

### Molecular signature of the microridge in zebrafish

**Figure 3:**
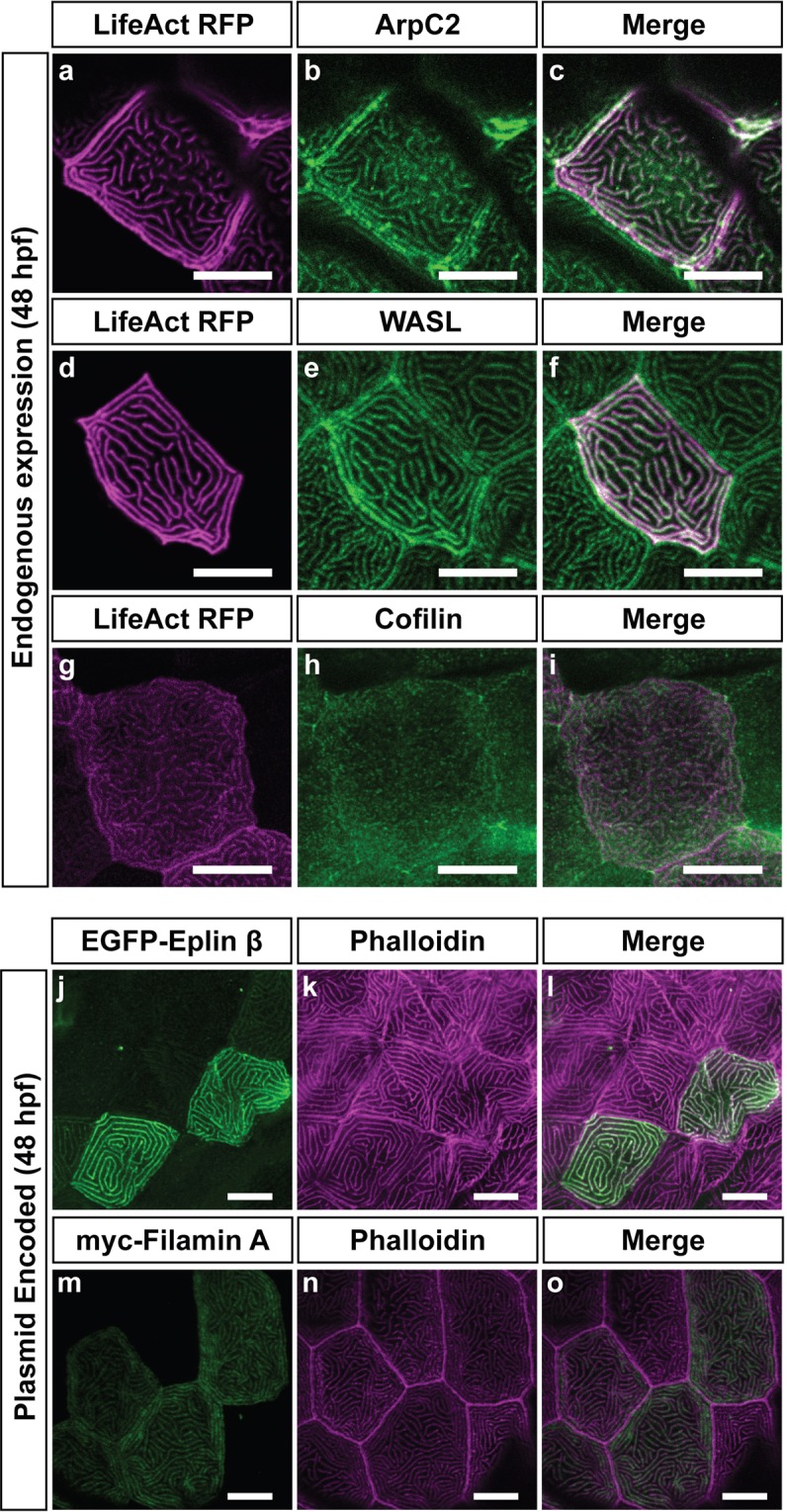
Localization of actin binding proteins at the microridge at 48 hpf. Immunolocalisation of ArpC2 (**b**), WASL (**e**), and Cofilin (**h**) at 48 hpf with LifeAct-RFP in magenta that marks F-actin (**a**,**d**,**g**) at 48 hpf. Respective merges are shown in (**c**,**f**,**i**). Plasmid encoded Eplin-β (**j**), and Filamin A (**m**) in green localize to microridges marked with phalloidin (**k,n**) in magenta in respective merges (**l**,**o**). Plasmids were injected at the 1 cell stage and expressed in a clonal fashion. While all other images are obtained from the head epidermis, Eplin images are taken on the flank. Scale bars are 10 µm.

As compared to microvilli^7,10^, the microridge is strikingly different in both morphology and F-actin organization. This prompted us to look at the molecular composition of microridges. Thus far, only a few actin-binding proteins are known to localize to the microridge. These include α-actinin, ezrin, myosin II, VASP and cortactin^9,11,13,34,39^. Since actin-binding proteins regulate and organize actin filaments into specific arrangements such as parallel or antiparallel bundles and branched networks^30,40^, we analyzed the localization of additional regulators of the actin cytoskeleton to microridges. We utilized two candidate-based strategies — a) antibody stainings to identify proteins endogenously present at the microridge and b) localization of plasmid encoded expression of tagged proteins. The immunolocalization analysis revealed that ArpC2, a component of the actin nucleator Arp2/3 complex^41^, localizes to microridges at 48 hpf (Fig. 3a–c). The nucleation promoting factor WASp-like (WASL) (Fig. 3d–f) and Cofilin (Fig. 3g–i), an actin severing and depolymerizing protein^41,42^, were also found to localize to the microridge though Cofilin showed relatively diffuse staining. As phalloidin staining was incompatible with the heat-based antigen retrieval staining protocols used for ArpC2, WASL and Cofilin, we used plasmid encoded LifeAct-RFP to mark microridges.

Our analysis using constructs of an EGFP tagged form of Eplin/Lima1 β (Fig. 3j–l) — an actin bundling protein found in stress fibers or at the adherens junction^43,44^ — and a myc tagged form of Filamin A (Fig. 3m–o) — an actin cross-linking protein found in branched structures like lamellipodia or membrane ruffles^45,46^ — revealed that the protein expressed from these constructs localized to microridges, labeled using phalloidin.

Since the activity of the Arp2/3 complex is essential for maintenance of microridges^13^, we asked whether there is a temporal correlation between the localization of the Arp2/3 complex, WASL and microridge formation. As the Arp2/3 complex is a bona fide component of branched actin networks^29,47^ and does not have a role in microvilli of the enterocyte^48^, we also reasoned that such an analysis would allow us to test the notion whether microridges are formed from microvilli. As a prerequisite for this analysis, we characterized the development of microridges starting from 50% epiboly (Fig. S5). We found that at 50% epiboly, F-actin punctae were present at the apical domain of EVL (enveloping layer) cells (Fig. S5a), which later give rise to the periderm^49^. Such punctae were visible till 12 hpf (Fig. S5b,c), after which microridges begin to elongate (Figs S5d–i, S6). To check if these punctae were associated with protrusions, we preformed SEM. We observed small protrusions at the periphery but not the center of the cell in most parts of the EVL at 9 hpf (Fig. S5j). By 18 hpf, microridges at the cell boundary were prominent and by 48 hpf most of the peridermal cell surface was covered with microridges (Fig. S5k,l).

**Figure 4:**
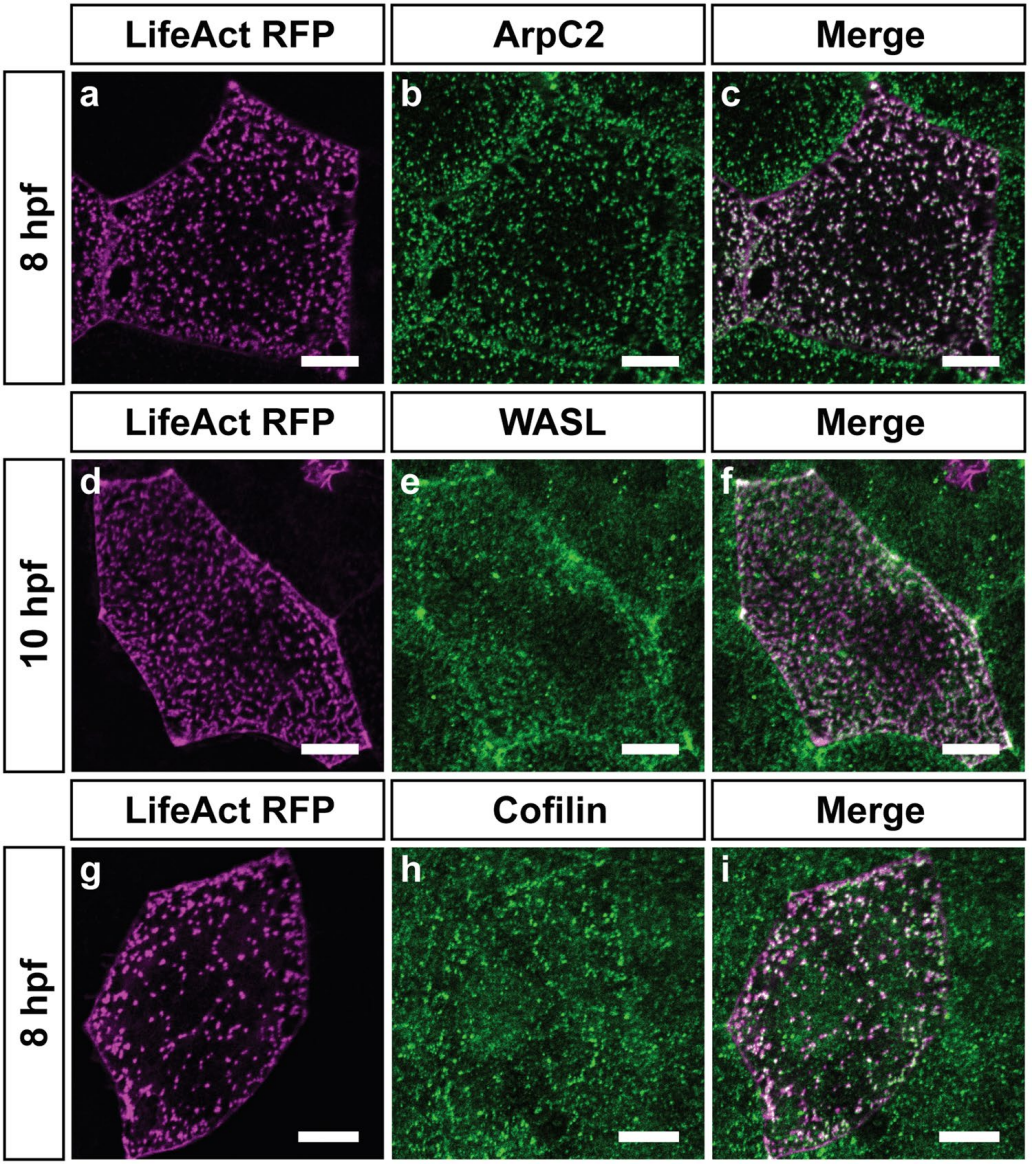
Immuno-localization of actin binding proteins at the apical domain prior to microridge elongation. Confocal scans of immunostainings performed for ArpC2 (**b**), WASL (**e**), and Cofilin (**h**) along with LifeAct-RFP in magenta (**a**,**d**,**g**) that marks F-actin at the time points mentioned. The antibody staining for ArpC2 (**b**), WASL (**e**) and Cofilin (**h**) are shown in green along with respective merges in (**c**,**f**,**i**). Scale bars are 10 µm.

**Figure 5:**
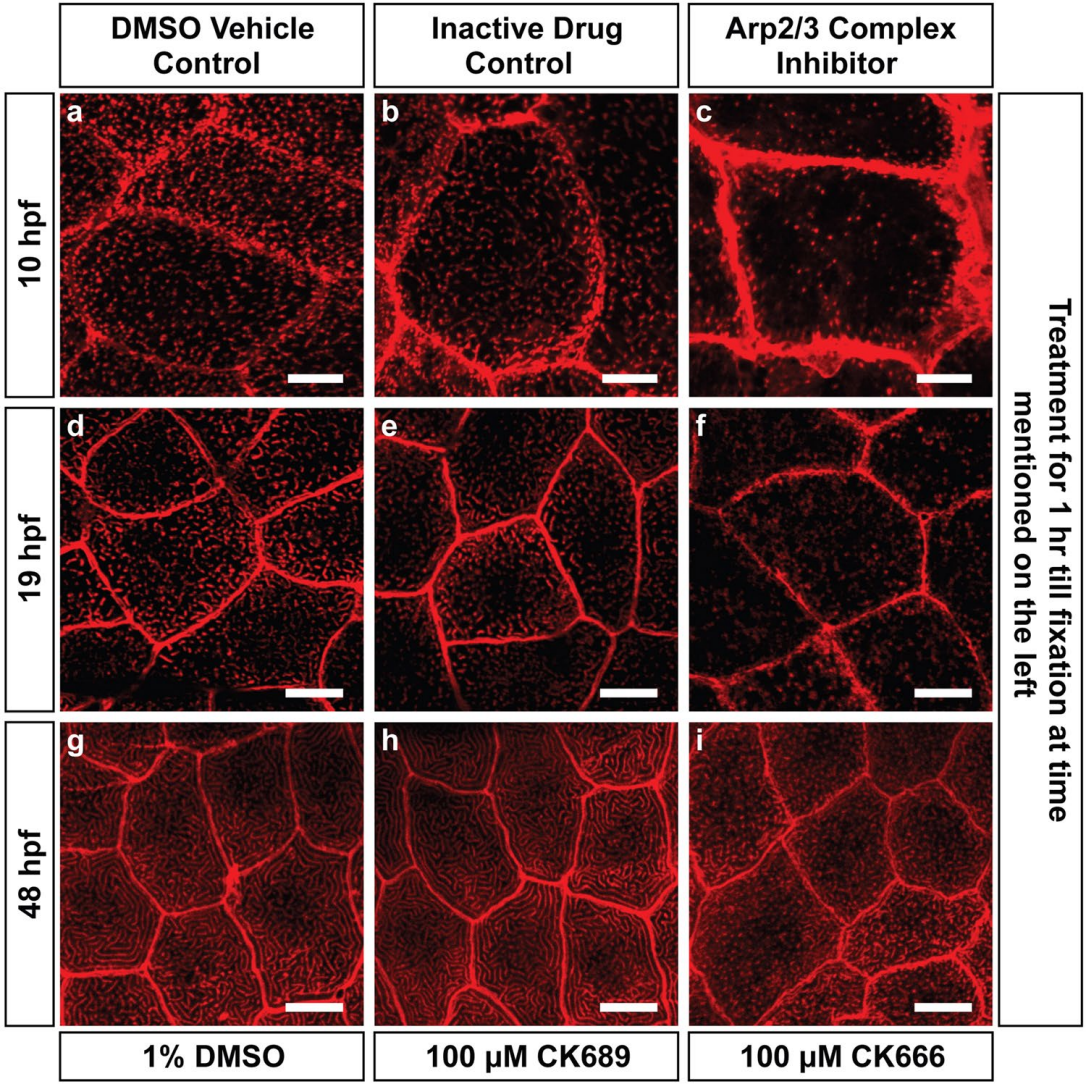
Arp2/3 complex activity is required for microridge formation and maintenance. Confocal microscopy analyses of phalloidin stained embryos and larvae (**a**–**i**) treated with 1% DMSO (**a**,**d**,**g**), 100 µM CK689 (inactive drug control; **b**,**e**,**h**) and 100 µM CK666 (Arp2/3 complex inhibitor; **c**,**f**,**i**) for 1 hour from 9–10 hpf (**a**–**c**), 18–19 hpf (**d**–**f**) and 47–48 hpf (**g**–**i**). In all cases, inhibition resulted in a breakdown of microridges or a decrease in phalloidin punctae relative to controls. Images from 19 and 48 hpf are acquired from the head epidermis. Scale bar are equivalent to 10 µm.

While ArpC2 (Fig. 4a–c) and Cofilin (Fig. 4g–i) colocalized well with F-actin punctae between 8–10 hpf, WASL localized in a diffused manner and partially colocalized to F-Actin (Fig. 4d–f). Inhibition of the Arp2/3 complex by CK666^50,51^ at 9 hpf for one hour followed by qualitative analysis revealed a stark reduction in F-actin punctae (Figs 5a–c, S7a–c,j), inhibition at 18 hpf prevented the formation of microridges (Figs 5d–f, S7d–f,j) and at 47 hpf resulted in their breakdown (Figs 5g–i, S7g–j), indicating that the Arp2/3 complex is important for both the formation and maintenance of microridges and punctae. Since keratins contribute to the terminal web, we also investigated if a temporal link exists between the formation of the keratin cytoskeleton and microridges. Keratin levels, analyzed by AE1/AE3 antibody staining, were relatively low at 9 hpf, increasing over time as monitored till 15 hpf (Fig. S8).

Thus, bona fide markers of branched actin networks such as the Arp2/3 complex, WASL and Filamin, as well as the actin-bundling protein Eplin, localize to microridges. Although Filamin and Eplin are of exogenous origin, their localization to microridges indicates their ability to bind to F-actin underlying the microridges. The Arp2/3 complex is essential for the formation or maintenance of actin punctae and microridges. At all time points analyzed, actin structures contained the Arp2/3 complex. Therefore, microridges form from actin punctae, presumably having branched configuration, and not from bundle based microvilli.

### Physiological relevance of microridges

The widespread occurrence of microridges on mucosal epithelial tissues has led researchers to propose that microridges function in retention and/or distribution of mucus^9,14,16,27^. The developing zebrafish embryo is covered by a glycan layer as early as the epiboly stages^5^, making it an excellent model to study the role of microridges in maintaining the organization of the glycan layer.

**Figure 6:**
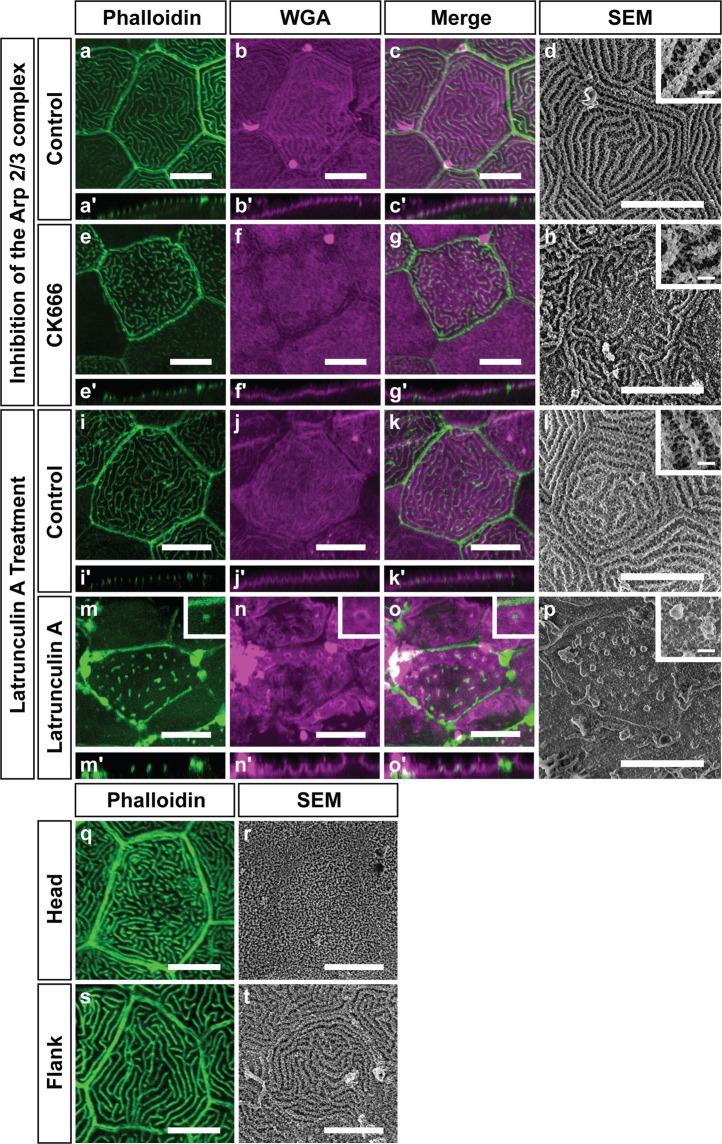
Microridges play a role in the organization of the glycan layer. Effect of the inhibition of the Arp2/3 complex on the glycan layer (**a**–**h**). Confocal micrographs of Phalloidin stainings (**a,e**), WGA stainings (**b,f**), merges (**c**,**g**) and SEM (**d**,**h**) of animals treated with 1% DMSO as vehicle control (**a-d**) or 100 µM CK666 (**e**–**h**) and respective orthogonal sections below the images (a’- c’,e’-g’). Effect of Latrunculin A treatment on the glycan layer (**i–p**). Phalloidin stainings  (**i,m**), WGA stainings **(j,n**) and merges (**k,o**) of animals treated with 1% DMSO as vehicle control **(i–k**) or 2 µM Latrunculin A (**m–o**) and respective orthogonal sections below the images (i’- k’; m’- o’). Insets in m-o show a microridge remnant (phalloidin, green) surrounded by the glycan layer (WGA staining, magenta). SEM using Alcian blue and Lysine fixation of 1% DMSO as vehicle control (**l**) or 2 µM Latrunculin A (**p**). The insets in d,h,l,p show higher magnification SEM images of their respective treatments. All of these images are acquired on the flank at 48 hpf. Differences in the glycan layer organization in different parts of the animal (**q**–**t**). Phalloidin staining (**q**,**s**) and SEM (**r**,**t**) of the head (**q**,**r**) and flank (**s**,**t**) peridermal cells at 48 hpf. Note that microridges on the head are relatively densely packed with a flat glycan layer as compared to the flank. Scale bars in the insets in d,h,l,p are equal to 1 µm and in others to 10 µm.

In order to visualize the glycan layer with a confocal microscope, we used a fluorescently tagged wheat germ agglutinin (WGA) lectin^39^. This approach revealed that the glycan layer was arranged around microridges (Fig. 6). To assess if microridges are important for the organization of the glycan layer, we used chemical inhibitors to disrupt the actin cytoskeleton and consequently the microridge pattern. While in control embryos, the WGA fluorescence followed the microridge pattern (Fig. 6a–c), in the CK666 treated embryos the WGA fluorescence was comparatively more uniform suggesting a mild effect on the organization of the glycan layer (Fig. 6e–g). SEM analysis on CK666 treated samples did not reveal a major change in glycan organization, again pointing to the mild effect of CK666 on the glycan layer (Fig. 6d,h). Similar to previous observations^13,34^, we achieved a severe perturbation of microridges using the actin monomer binding drug Latrunculin A (Lat A) (Coue *et al*., 1987). LatA treatment resulted in the decreased density and length of microridges (Fig. 6i–p). We analyzed the ventral region of the flank where the effect of LatA on microridges was most severe. As compared to the control (Fig. 6i–k), the LatA treated samples showed more intense WGA staining around the microridge remnants indicating accumulation of glycans around them (Fig. 6m–o). SEM analysis revealed that the glycan layer persisted in the troughs between microridges at a lower density. However, it was clearly enriched around the microridge remnants (Fig. 6l,p).

During our analyses, we observed that microridge patterns differed on the flank (Fig. 6s) from those on the head (Fig. 6q), with a higher packing density of microridges on the head. SEM analysis revealed that the glycan layer coats microridges on the head in a sheet-like fashion without revealing their underlying pattern (Fig. 6r). In contrast, the glycan layer on the flank follows the pattern of underlying microridges, with glycans enriched on microridges with apparent gaps in between (Fig. 6t). This further indicates that microridges have the ability to retain glycans in their close proximity.

In conclusion, peridermal cells on the zebrafish larval epidermis are covered with a thick glycan layer. The organization of this layer relies on the underlying microridges.

## Discussion

The apical surface is functionally the most important part of an epithelial tissue. It is decorated with actin based projections and a glycan layer. Laterally long protrusions such as microridges have been known to morphologists for over 50 years now but have not been well studied. The zebrafish periderm, which is almost completely covered with microridges, offers an ideal system to study such protrusions and their role in glycan biology^13,34^. Here, we used immunofluorescence, EM tomography and SEM analysis to understand how actin microridges form, are maintained and whether they function in glycan organization.

**Figure 7:**
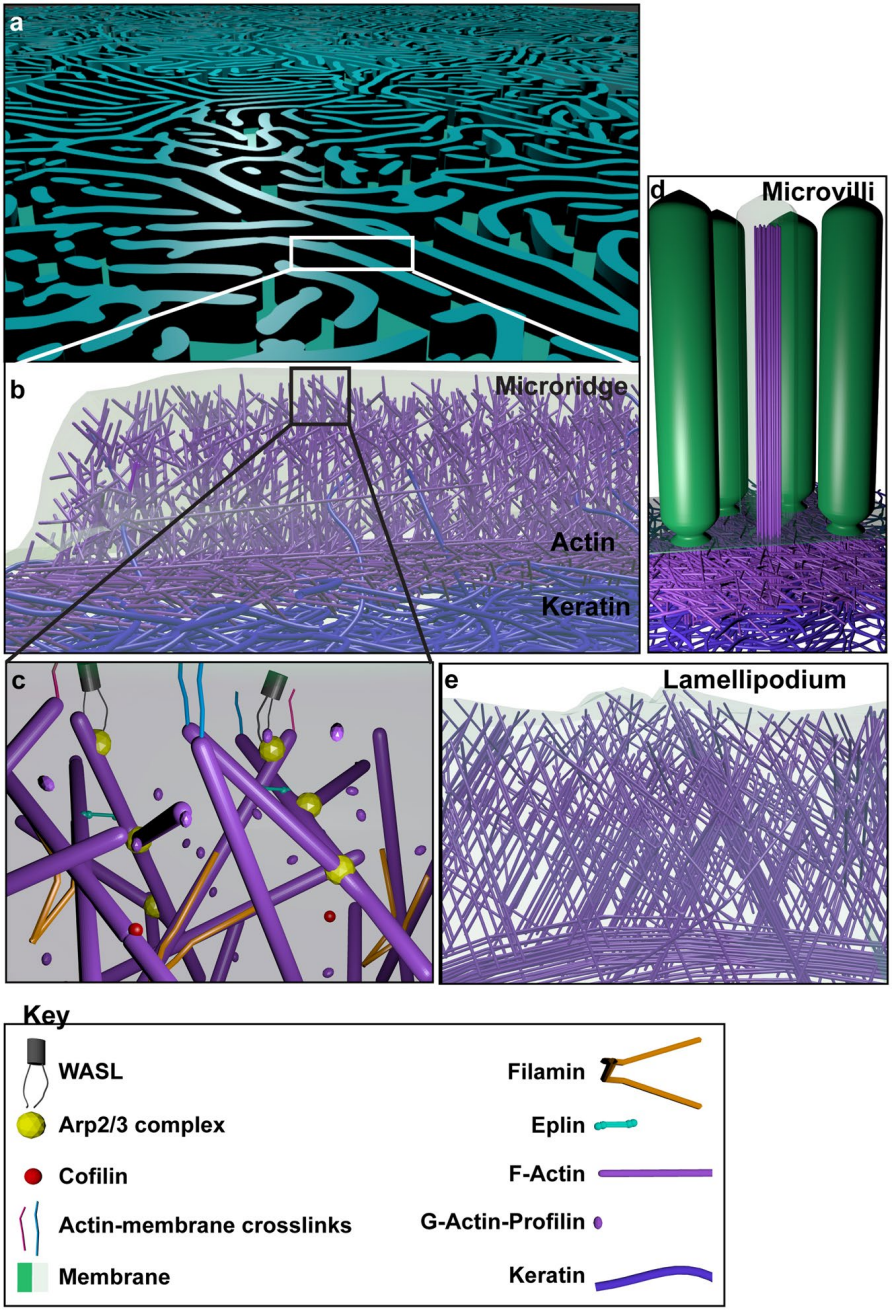
A schematic representation of the actin microridge based on EM and immunolocalization analyses. The actin microridge is a laterally long protrusion (**a**). It consists of a branched network of actin with an underlying sub-protrusive zone of actin (magenta) and keratin (blue) (**b**). Occasionally, keratin filaments also enter the protrusion (**b**). The actin microridge protrusion itself consists of various molecules (**c**), many of which associate with branched networks. The sub-protrusive zone of the actin microridge resembles the sub-protrusive terminal web of brush border microvilli (**d**), which are protrusions made up of bundled actin with a terminal web of actin and keratin. On the other hand, the branched actin architecture of the microridge protrusion has similarities with the lamellipodial protrusion (**e**).

Our ultra-structural analyses presented here indicate that the microridge is largely made up of a network of actin supported by an underlying keratin cytoskeleton. This organization is in fact similar to that observed in lamellipodia, wherein the lamellipodial network meets the transverse actin arc^52^. Our results in zebrafish larvae at 48 hpf are consistent with Uehara *et al*., who have shown that microridges in carp oral mucosa exhibit actin filaments oriented randomly^20^. However, unlike our ET analysis, this earlier analysis was done with detergent extracted samples and hence did not reflect the complexity of the network in its entirety. Our structural analysis is largely in agreement with the fact that regulators of branched actin conformation - the Arp2/3 complex and WASL - localize to the microridges and that Arp2/3 complex function is required in forming and maintaining microridges. In this sense, microridges can be thought of as several stable lamellipodium-like protrusions organized in a labyrinthine manner on the apical surface of cells (Fig. 7a–c). Both SEM and tomographic data show that the microridge cytoskeleton is contiguous with the cortex and keratin network, suggesting that the organization of this layer would influence the microridge pattern (Fig. 7b). Curiously, in lamellipodia the sub-protrusive lamella appears to be primarily actin based^52,53^, whereas in microridges it is formed of both actin and keratin, similar to microvilli (Fig. 7b,d,e)^54^. In fact, keratin filaments from the sub-protrusive zone also enter microridges, possibly offering additional structural support (Fig. 7b).

Intriguingly, we also observed localization of exogenously expressed bundling proteins Fascin (Khandekar and Sonawane, unpublished observations) and Eplin, to microridges. It is possible that though the F-actin network supporting microridges is largely controlled by regulators of branched actin, there might be a small but significant contribution from regulators of bundled actin. This is not surprising given the fact that structures formed of bundled actin, such as microspikes and filopodia, are associated with lamellipodia^55^. Alternatively, such components of bundled actin may be present to enable the transformation of the microridge actin cytoskeleton conformation from a largely branched to a combination of branched and bundled actin, under certain developmental or physiological conditions. Indeed, Bereiter-Hahn and co-workers have shown that microridges on the scale epidermis of adult guppy females exhibit a few actin bundles spaced along the length of the microridges^11^. Furthermore, Depasquale suggests a potential model, which consists of branched actin with bundled actin cores spaced along the length of the microridge^9^. Therefore, it is possible that epidermal microridges of the later developmental stages contain prominent bundles along with branched actin.

During development, the Arp2/3 complex localizes to F-actin punctae as well as microridges. In addition, the function of the Arp2/3 complex is important for the formation of actin punctae suggesting that they are formed of branched actin. Such punctae might fuse to form microridges^34^. However, the functional association of these punctae with the Arp2/3 complex and the fact that we did not observe the microvillar tip-complexes along the microridge, suggest that microridges on embryonic peridermal cells in zebrafish are unlikely to be formed from the fusion of bundled actin containing microvilli as shown for A6 cells^33^. In light of our findings the actin conformation as well as the presence or absence of the Arp2/3 complex in the microvilli of A6 cells needs to be analyzed. In contrast to the Arp2/3 complex, which is associated with punctae even at 10 hpf, keratin levels in the tissue are relatively low at early time points and rise concomitant with the growth of microridges. This increase in keratin levels might be linked to the specification of the periderm and could therefore be a trigger for microridge formation.

The ubiquitous presence of microridges during vertebrate evolution suggests an important function for them. Microridges have been proposed to have various important functions, the most common one being the retention of mucus. Indeed, disruption of microridges by LatA, visibly affected the glycan layer. From both confocal and SEM data, it is clear that glycans are preferentially retained around the spot-like microridge remnants. Although, Arp2/3 complex inhibition had only a mild effect on the glycan layer, our TEM, SEM and WGA staining data as a whole suggests that glycans are enriched around microridges. WGA staining also follows the microridge pattern in the case of koi epidermal cells^39^. It is thus likely that the distance between microridges is an important factor in the organization of the glycan layer, which is corroborated by the fact that the glycan layer on the head, which has closely packed microridges, exhibits a dense and uniform organization as compared to that on the flank, where microridges are spaced apart.

In summary, the actin microridge is a unique protrusion that has potential evolutionary origin from and structural homology to the leading edge of migrating cells, with an underlying actin and keratin containing sub-protrusive zone and functions in the organization of the overlying glycan layer. It will serve as an excellent model to further probe the role and regulation of actin networks *in vivo*.

## Materials and Methods

### Fish strains

For microridge experiments, the zebrafish *Tübingen* (*Tü*) wild-type strain was used. For the tomography of microvilli, a wild-type sibling fish of the *goosepimples* strain *gsp*^*NSO42*^ was used. For zebrafish maintenance and experimentation, the guidelines recommended by the Committee for the Purpose of Control and Supervision of Experiments on Animals (CPCSEA), Govt. of India, and approved by the institutional animal ethics committee vide the approval TIFR/IAEC/2017-11, were followed.

### Plasmid injections

Plasmids, EGFP-EPLIN β - a gift from Elizabeth Luna (Addgene plasmid # 40948), pcDNA3-myc-FLNa WT - a gift from John Blenis (Addgene plasmid # 8982)^56^, LifeAct-TagRFP (Ibidi; 60102)^57^, were purified using Invitrogen’s plasmid mini-prep kit and dissolved in autoclaved Milli-Q water (Millipore) or nuclease free water. Plasmids were injected at a concentration of 40 ng/µl in autoclaved Milli-Q water at the 1 cell stage.

### Inhibitor treatments

CK666 (182515, Calbiochem), its inactive control CK689 (182517, Calbiochem) and Latrunculin A (L5163, Sigma) were dissolved in dimethylsulfoxide (DMSO). For treatment with inhibitors, 10 dechorionated embryos/larvae were added in 1 ml E3 without methylene blue to a well of a 12 well plate and 1 ml of 2x inhibitor (containing final DMSO concentration of 1%) or vehicle (1% DMSO - final concentration) as control in E3 without methylene blue was added to it and mixed gently with a pipette. CK666 and CK689 treatments (100 µM) were carried out for 1 h whereas Latrunculin treatment (2 µM) was performed for 30 min which is similar to a previous study^34^.

### Immunohistology and imaging

For immunostainings, a previous method was employed^58^, larvae were fixed in 4% PFA for 30 mins at room temperature (RT) and then overnight (O/N) at 4°C. They were washed in phosphate buffered saline (PBS) and permeabilized with PBS containing 0.8% Triton X-100 (PBSTx), blocked in 10% normal goat serum (NGS; 005-000-121, Jackson Immuno Research Labs) in PBSTx for 3 h, incubated with primary antibody in 1% NGS in PBSTx for 3 h to O/N, washed with PBSTx 5 times for 30 min each, incubated with secondary antibody in 1% NGS in PBS for 3–4 h, followed by 5 washes in PBSTx each for 10 min. Samples were then fixed for 30 min or O/N in PFA, washed twice with PBS and upgraded in 30%, 50%, 70% glycerol in PBS.

For ArpC2, WASL and Cofilin stainings heat induced antigen retrieval was required (based on http://www.ihcworld.com/_protocols/epitope_retrieval/citrate_buffer.htm). The samples were equilibrated in Tris-Cl (150 mM, pH 9.0, for ArpC2 and WASL) or sodium citrate buffer (10 mM sodium citrate, 0.05% Tween-20, pH 6.0, for Cofilin) for 5 min. Subsequently, the samples were incubated with fresh Tris-Cl or sodium citrate buffer at 70 °C for 20 min. The samples were then allowed to reach room temperature, washed in PBSTx, and processed for immunostaining from the blocking step as above.

For phalloidin staining, larvae were fixed in 4% PFA as before or for 3 h at RT. The larvae were then washed 5 times in PBS for 10 min each, incubated in 1:40 phalloidin for 3–4 h followed by 5 washes in PBS for 10 min each and upgradation in glycerol in PBS. Phalloidin rhodamine (R415, Molecular Probes) or Alexa Fluor 488-phalloidin (A12379, Molecular probes) were used. When phalloidin and antibody staining were performed together, phalloidin (1:400 in PBSTx) was used at the secondary antibody step.

For α-Tubulin staining, larvae were fixed in ice cold Dent’s fixative (80% methanol, 20% DMSO) on ice for 30 mins then at −20 °C for 2 hours to O/N. Larvae were then downgraded in a methanol series, 100%, 70%, 50%, 30% methanol in PBS, followed by 2 washes with PBS and immunostainings.

Primary antibodies used in this study include mouse anti-GFP (clone 12A6, DSHB; 1:100), rabbit anti-GFP (TP401, Torrey Pines Biolabs; 1:200), anti-Cofilin (SAB4300577, Sigma-Aldrich; 1:100), anti-ArpC2 (HPA008352, Sigma Prestige; 1:100), anti-Wasl (HPA005750, Sigma Prestige; 1:100), anti-Myc (9E 10-s, DSHB, 9E 10 was deposited to the DSHB by Bishop, J.M; 1:100), anti-pan Cytokeratin [AE1/AE3] (ab27988, Abcam; 1:100), anti-α-Tubulin (T9026, Sigma; 1:100). The secondary antibodies and their dilutions were as follows: anti-mouse Alexa 488-conjugated (A-11029, Invitrogen; 1:250), anti-rabbit Alexa 488-conjugated (A11034, Invitrogen; 1:250), anti-rabbit Alexa 546-conjugated (A-11035, Invitrogen; 1:250), goat anti-rabbit Cy3 (111-165-144, Jackson ImmunoResearch; 1:750), goat anti-rabbit Cy5 (111-175-144, Jackson ImmunoResearch; 1:750), goat anti-mouse Cy3 (115-165-146; Jackson ImmunoResearch; 1:750), goat anti-mouse Cy5 (115-175-146, Jackson ImmunoResearch; 1:750). DSHB-GFP-12A6 was deposited to the DSHB by DSHB (DSHB Hybridoma Product DSHB-GFP-12A6).

Imaging was carried out using the Zeiss LSM 510 Meta or Zeiss LSM 710 with EC Plan-Neofluar 40X/1.30 oil objective at 2x zoom or Plan-Apochromat 63x/1.40 oil objective at 1.5x zoom (Zeiss). 1024 × 1024 image dimensions were used, with an averaging of 4.

For localization experiments, animals were either plasmid injected (EGFP-EPLIN β, pcDNA3-myc-FLNa WT, LifeAct-TagRFP) or uninjected (Keratin and α-Tubulin). Number of sets and total number of animals are indicated in brackets as (sets; animals). Images are representative for the following: For ArpC2, WASL and Cofilin (2; 12), for Eplin-β (2; 10), for Filamin-A (1; 3), Keratin (48 hpf) (2; 10), α-Tubulin (2; 10).

### Estimation of keratin intensity

Samples for keratin estimation were imaged on the Zeiss LSM 710, at 12-bit depth. Images were analyzed in FIJI^59^. A circle with a diameter of 10 µm was made at a place in the cell at one slice below the ridge slice and its mean intensity was obtained. It was ensured that no circle had any point at saturation within it.

### Electron Microscopy (EM) and EM-tomography

For EM, larvae at 48 hpf were fixed with two protocols. The first is based on Tilney and Tilney^60^, with slight modifications. Briefly, larvae were fixed for 30 minutes at 4 °C in 1% OsO_4_ (60H0150, Sigma) and 1% GA (11614, Electron Microscopy Sciences - EMS) in 0.1 M Phosphate buffer (PB) at pH 6.2. They were washed in ice cold Milli-Q water, 3 times for 5 minutes each then en bloc stained in 0.5% Uranyl acetate (22400, EMS) for 1 hour at RT, dehydrated in 30%, 50%, 70%, 90%, 100%, 100%, 100% acetone series for 5 min each. The samples were then brought in acetone: Epon-Araldite (1:1) for 30 min followed by acetone: Epon-Araldite (1:2) for 30 min, O/N in Epon-Araldite (13940, EMS) and then placed in moulds and cured at 60°C.

For the other protocol, samples were fixed in 2.5% GA and 1% tannic acid (21710, EMS) in 0.1% PB at pH 7.2 for 45 min and then O/N in just 2.5% GA in the same buffer, washed in Milli-Q water 6 times, en block stained with 0.5% uranyl acetate for 1 hour at RT and then dehydrated and embedded in the same way as above.

Protocol for the intestinal sample was as described previously^38^. Briefly, a 6 dpf wild-type sibling was fixed in 2.5% each PFA and GA for 30 mins at RT and then at 4 °C O/N. Then washed with PB, fixed with 1% OsO_4_, en bloc stained with uranyl acetate, dehydrated in ethanol and embedded in Epon-Araldite.

For standard EM, 70–100 nm sections cut with a diamond knife (DiATOME) were collected on formvar coated slot or mesh grids or uncoated mesh grids and post stained with uranyl acetate and lead citrate and imaged on a Tecnai 12 (FEI) microscope. For EM tomography, 300 nm sections were collected on formvar coated slot grids, post stained with uranyl acetate, lead citrate and then with colloidal gold to add fiducials to both sides of the grid (10 nm gold nanoparticles, Sigma-Aldrich) and further carbon coated on both sides.

Tomograms were acquired and reconstructed as previously described^61^ with modifications. Electron tomograms were collected on a Tecnai F-30 (FEI) TEM at 300 kV with a 2048 × 2048 Gatan CCD camera. Tilt series were collected in dual tilt axis geometry and maximum tilt range of 64–65° and tilt steps of 1° in an automated manner using SerialEM software^62^. For the large tomogram (Fig. 1a–i) a montage of 9 images was collected. The IMOD software package^63^ was used to reconstruct and visualize the tomograms.

### EM tomogram segmentation

The EM images were de-noised using a low pass Gaussian smoothening followed by the Perona-Malik anisotropic diffusion method^64^. These steps ensured image smoothening keeping region boundaries and small structures within the image unperturbed. Subsequently, to analyze the local behavior, 2D Hessian images were constructed as explained previously^65^. We exploited the eigenvalues of the Hessian to extract actin structures within each image^66^. The higher of the eigenvalues encompassed the salient features of the actin structures within the microridges. The eigenvalue images were binarised and similar pixels were connected in depth to obtain an n-dimensional image of the tomogram.

On the 3D binarised image, three morphological operations were performed consecutively using the *bwmorph3* MATLAB function. Interior individual voxels within connected regions were filled-in with 1’s by using a ‘fill’ operation subsequently followed by a ‘clean’ operation to remove isolated/disconnected voxels and finally a ‘majority’ operation was performed. The volume property of each segmented microridge (branch points with their emanating branches) was computed using *regionprops3* MATLAB function. Only segmented volumes >265 nm^3^ were included for further analysis. The 3D segmented image sub-volumes were skeletonized using *bwskel* MATLAB function and their branch points and endpoints were identified using the *bwmorph3* MATLAB function.

The branch lengths and angles between neighboring branches were computed on the skeletonized images. For this, the 3D skeletonized segmented image was subtracted from the branch-point image to obtain individual branches of an inter-connected microridge. The *regionprops3* MATLAB function was used to obtain voxel list of points that makeup a branch and their corresponding eigenvectors and eigenvalues. Using a custom-built MATLAB program, the branch lengths were computed based on Euclidean distances between subsequent 3D voxel points of a branch. Our visual inspection revealed that the branches followed a curvilinear path rather than straight lines. We obtained the shortest path to connect all voxels of a branch. We computed Euclidean distances between all pairs of 3D voxel points of a branch and averaged two minimums of distances for each voxel point. The sum of such distances then provided the shortest branch path and hence their lengths.

For computation of angles between neighboring branches of microridges, all pairs of branches emanating from the same branch-points were considered iteratively for each segmented structure. The eigenvector corresponding to the highest eigenvalue was taken as the branch direction. The angle ($\varphi$) between two branches emanating from a common branch point were computed using:

1 $$\varphi ={\cos }^{-1}(\frac{\overrightarrow{{v}_{1}}.\overrightarrow{{v}_{2}}}{\Vert \mathop{\to }\limits_{{v}_{1}}\Vert \Vert \mathop{\to }\limits_{{v}_{2}}\Vert })\,$$

where ${v}_{1}$ and ${v}_{2}\,\,$are the eigenvectors corresponding to the highest eigen values of two branches emanating from the same  branch point.

### Generation of synthetic images mimicking actin microridges in EM tomogram for validation of segmentation algorithm

The segmentation algorithm was verified using artificial images.

Artificial images were generated by initiating a simple branching fractal pattern^67^. Fractal models have been previously used for studying branching lung morphogenesis^68^ and human mammary lobule^69^. An interconnected pattern in 3D consisting of 6 branches with common starting and ending vertices (Fig. S3a) was produced. The interconnected branches shared two branch points with 3 branches emanating from a single branch point and 4 branches emanating from the other. Next, 30 intermediate points were generated using a linear interpolation technique for each branch using the MATLAB interpac function^70^ (Fig. S3b). In order to introduce curvature within each branch, any three of the trigonometric functions 0.5 × (sin/−sin/cos/−cos) were randomly chosen with randomly chosen angles between 0 and 2π radians, and were added to every second 3D branch point excluding the two starting and the two endpoints of each branch. Smooth curvilinear 3D points for each branch was obtained after spline interpolation (MATLAB interpac function^70^) followed by a smoothening function (smoothdata in MATLAB; Fig. S3c).

A two-dimensional region of the EM data at a single depth without any filaments was used to estimate noise within the synthetic images. Raw histogram of pixels within such a region of varying sizes typically obeyed a normal distribution (Fig. S3d). Using the computed variance and mean of the pixels from within the real image, a three-dimensional noise matrix with normal distribution was generated of size 80 × 80 × 80 pixels.

A three-dimensional matrix of similar size as that of the noise image with all values equal to ones was produced. A Gaussian function was then used to convert the 3D set of points of the branched lines into a 3D image given by,

2 $$I(\overrightarrow{x})=\,\sum _{c}\,A\,exp(-\frac{|\overrightarrow{x}-\overrightarrow{{r}_{c}}|}{{\sigma }^{2}})$$

where Α = 25, σ = 0.7, x is the pixel (a.u.) and r_c_ is the parameterization of the 3D branches.

Noise and image matrices were added to produce an image with 3D branch images with background noise (80 × 80 × 80 pixels). Blank images with only background noise were removed and the 3D-matrix was set to ‘uint8’ type to produce an 8-bit three-dimensional image of size 80 × 80 × 26 voxels with a spacing of 0.707 nm in each direction (Fig. S3e). Image segmentation with same parameters as in Fig. 2 was used to segment and compute the branch statistics within the artificial images (Fig. S3f,g). Because a random trigonometric function was used to produce the curvature, for each run of the algorithm, a slightly different topology could be generated, with the same starting fractal pattern of 6 branches. This allowed varying branch length statistics and branch angles as observed within the real EM data. Figure S3h–j shows the segmented volume of 6 branches and their skeleton images with highlighted branch points and endpoints respectively.

To measure error rates of the segmentation algorithms in synthetic images, the branch lengths obtained after automated segmentation were compared with ground truth lengths. Synthetic images were manually segmented using the IMOD software package^63^ to determine the true branch lengths. The algorithm was evaluated to have segmented correctly when the absolute difference between true branch length (manual segmentation) and the length obtained after segmentation with the proposed algorithm were within an empirically determined threshold value of 3.5 nm. Accordingly, misses were scored when such absolute differences were larger than the threshold value.

The correctly determined lengths by the algorithm were counted as B_DL_ and the sensitivity was defined as B_DL_/B_TL_, where B_TL_ is the total number of branches in the synthetic images. The number of segmented branches was found to have a match with that obtained after manual segmentation.

### Scanning electron microscopy on intact and detergent extracted samples

For detergent extraction, a previously published protocol^71^ with a few modifications was used. Larvae were dechorionated and detergent extracted in 2 ml tubes with 500 µl PEM buffer (100 mM PIPES (free acid) pH 6.9, 1 mM MgCl_2_ and 1 mM EGTA) containing 2% PEG (M.Wt. 35,000) (81310, Sigma), 1% TritonX-100 and 2 µM phalloidin (P3457, Invitrogen) for 30 seconds. To the same tube, without removing any solution, 500 µl 0.4% GA in PEM containing 0.5% TritonX-100 was added and incubated for 2.5 min. Most of the solution was removed (at no point was the sample allowed to come in contact with the air-solution interface) and 1 ml of 2% TritonX-100 in PEM containing 2 µM phalloidin was added and the samples were incubated for 7 min followed by 3 washes with PEM containing 2 µM phalloidin for 1 min each. The samples were fixed in 2% GA in PEM (without phalloidin) for 20 min at RT and then O/N at 4°C.

The samples were washed thrice with distilled water for 5 min each, treated with a 0.2% tannic acid solution in distilled water for 20 min, washed again 5 times with distilled water for 5 min each and then treated with 0.4% uranyl acetate in distilled water for 1 h at RT. Samples were then washed 3 times with distilled water and dehydrated in an acetone series (30%, 50%, 60%, 70%, 80%, 90%, 100%, 100%, 100% for 5 min each) and 1:1 Hexamethyldisilazane (440191, Sigma): Acetone for 5 min. Subsequently, neat Hexamethyldisilazane treatment was carried out for 5 min and again for 10 min followed by removal of the excess Hexamethyldisilazane. The samples were picked with filter paper and left to air dry for 2–4 h. Post drying samples were placed on a stub with carbon tape, sputter coated with gold for 1 min and imaged with a Zeiss Gemini SEM. For detergent extracted samples, SEM was performed on 3 animals in total from 2 sets.

For SEM on non-detergent extracted larvae fixation was done in 2.5% gluteraldehyde for 30 min at RT and then O/N at 4 °C. Then either the protocol described above for detergent extraction samples from the washes and tannic acid step or an OTOTO protocol was followed with changes^37^. For OTOTO, the samples were first stained with OsO_4 _(O) for 15 min, washed 3 times, stained with saturated aqueous Thiocarbohydrazide (T) (223220, Sigma-Aldrich) for 15 min and washed 3 times. This was repeated till the O-T-O-T-O steps were completed and then dehydrated with acetone as above for the detergent extracted samples, without sputter coating.

### Preservation and imaging of the glycan layer

For imaging by light microscopy, larvae at 48 hpf were fixed with 75 mM Lysine (124828, SRL), 0.1 M Cacodylate buffer (11655, EMS), 2% PFA (103999, Merck), 2.5% gluteraldehyde (11614, EMS). The PFA and gluteraldehyde were added together just prior to use. The larvae were fixed for 22–24 h at RT. The samples were then washed 4–5 times with PBS. They were then incubated with WGA-594 (W11262, Invitrogen; 1:200) along with Phalloidin-Alexa488 in PBS for 3–4 h at RT, followed by 5 washes with PBS (5–10 min each). Finally, the samples were serially upgraded into 70% glycerol and mounted for confocal microscopy.

For imaging by SEM, a protocol similar to Fischer *et al*.^37^ was used. Larvae at 48 hpf were fixed with a cocktail containing 75 mM Lysine (124828, SRL), 0.1 M Cacodylate buffer (11655, EMS), 2% PFA (103999, Merck), 2.5% gluteraldehyde (11614, EMS), 0.075% Alcian Blue (48261, SRL) for 22–24 h at RT. Then the protocol for OTOTO staining was followed including the dehydration and imaging steps.

For TEM, the above SEM protocol was used for sample preparation, including the OTOTO steps, then the samples were dehydrated, embedded and processed for EM analysis as above and imaged in a Zeiss Libra 120 microscope at 120 kV.

Images are representative for the following: SEM (2 sets; 6 animals in total), WGA (2 sets; 12 animals in total).

### Estimation of microridge parameters

For estimation of microridge height, microridges that appeared to be straight were measured from their base to the tip manually in FIJI^59^ using TEM images. For estimation of microridge width, measurements were made roughly perpendicular to the microridge length manually in FIJI using SEM images, some microridges were estimated at multiple locations along their length.

For the estimation of mean microridge length, an in house FIJI macro based script was used, which will be described and published separately, but will be provided upon reasonable request. Briefly, interconnected microridges were split at their connection point^72^, and the length of the microridges was estimated. The mean microridge length, $\overline{{M}_{L}}$ per cell was given by

3 $$\overline{{M}_{L}}=\frac{{\sum }_{i=1}^{M}\,{m}_{i}}{M}$$

where ${m}_{i}$ is the length of individual microridges and M is the total number of microridges within a cell.

### Statistical analysis and image processing

Processing of fluorescence microscopy images was carried out using FIJI. Panels were made in ScientiFig^73^ and exported to Adobe Illustrator or directly made in Inkscape (Inkscape Project), statistical analysis and plot generation (using the ggplot2 package) was carried out using R in Rstudio^74–76^. Description of the boxplots — the line in the middle is the median, lower and upper box limits show the 1st quartile (Q1) and 3rd quartile (Q3) positions respectively, the whiskers extend 1.5 times the interquartile range or to the furthest point from the box limit, whichever is closer to the box limit and each point indicates a data point used in generating the boxplot^77^. Images for the model were created in Blender (Blender Foundation). Segmentation analysis, generation and validation of synthetic images were carried out using MATLAB R2018a-2018b.

## Supplementary information

Supplementary information

Video 1

Video 2

Video 3

Video 4

Video 5
