# Supplementary material for: Microridges are apical epithelial projections formed of F-actin networks that organize the glycan layer
Source: Sci Rep. 2019 Aug 21;9:12191. doi: 10.1038/s41598-019-48400-0 (PMC6704121; doi:10.1038/s41598-019-48400-0)
Supplement: Supplementary file 1 — Supplementary information [file 41598_2019_48400_MOESM1_ESM.pdf]

Microridges are apical epithelial projections formed of F-actin networks that organize the glycan layer

Clyde Savio Pinto[1], Ameya Khandekar[1], Rajasekaran Bhavna[1], Petra Kiesel[2], Gaia Pigino[2], Mahendra Sonawane[1,$]

[1]Department of Biological Sciences, Tata Institute of Fundamental Research, Colaba, Mumbai, India;

[2]Max Planck Institute of Molecular Cell Biology and Genetics, Dresden, Germany

Description of Videos

Video 1: An electron tomogram through the long axis of the microridge. Each frame represents a 0.7 nm thick section in the Z axis. The scale bar is 200 nm. The sample was prepared using GA and OsO_4. This is the same tomogram shown in Fig. 1c-h.

Video 2: An electron tomogram of a transverse section of a microridge at the cell junction. Each frame represents a 0.55 nm thick section in the Z axis. The scale bar is 200 nm. The sample was prepared using GA and OsO_4. This is the same tomogram shown in Fig. 1i-k.

Video 3: An electron tomogram at an angle through the transverse section of the microridge. Each frame represents a 0.55 nm thick section in the Z axis. The sample was prepared using GA and tannic acid but without OsO_4. The tannic acid makes actin filaments appear thicker than in the previous cases. The scale bar is 200 nm.

Video 4: Segmentation of an electron tomogram of a microridge reveals a network of actin. A segmented region shown in Fig. 2b,f of the tomogram in 3D. The Z-depth is indicated by the colourbar.

Video 5: An electron tomogram through an enterocyte of a 6 dpf wild-type sibling larva. Each frame represents a 0.7 nm thick section in the Z axis. The scale bar is 200 nm.

Supplementary figure legends

Figure S1. Ultrastructural analysis of the peridermal apical domain.

Scanning electron micrographs of the head periderm (a). SEM analysis shows that long microridges (black arrowheads in a) flank the tight junctions on either side of the cell margin. These junctional microridges may be discontinuous (white arrows in a). Quantification of microridge height and width (b) as measured by TEM and SEM, respectively. TEM (c) analysis in presence of Alcian Blue and Lysine revealed a thick layer of glycans present above microridges. Inset in c, which is a zoomed in version of the boxed region, shows glycans above and around the microridge. Normal TEM of microridges (d,e). A tight junction as shown by the bracket in d connects the two cells. Note the presence of a vesicle at the location shown by the white arrowhead in e.

Images are obtained from the head of 48 hpf larvae. Scale bar in a represents 10 µm, in c is 2 µm and in d,e are equal to 0.2 µm.

Figure S2. Actin network within the microridge after segmentation and reconstruction of an electron tomogram in Video 3

An EM image sub-volume of 1881×2003×300 voxels at equal spacing of 0.553nm in each direction was chosen and shown here at a depth of 85.7nm (a). The actin structures within the microridge shown in (b) is outlined by Higher Eigen value 2D matrix. Similar to Fig 2, for

illustration of the segmentation analysis, a cubic section of 55.3×97.8×43.1 nm3 (dotted cube in b) is cropped. Image binarization (in blue) - shown at 3 different depths - outlines the actin structures (c). The arrangement of actin meshwork is revealed by 3D reconstruction of actin (d) and corresponding 3D skeleton image (e). Depth is indicated by a colorbar. An example of single rendered actin structure (f) along with corresponding skeleton image (g), showing branch lengths (in nm) and angles (degree) between neighboring branches. Short branches with fewer voxels (<11) were considered as noise removed from the image and excluded from computation of branch lengths and inter-branch angles. Branch points (green) and endpoints (black) are highlighted on the 3D skeleton images. Within this sub-volume, branch lengths (h) were within the range of 10-20nm. Angles between neighboring branches emanating from a common branch-point were found to be between -90° to +90°, predominantly in the range of ±60° to ±90° (i). This sample was prepared using GA and tannic acid but without OsO4.

Figure S3. Generation of synthetic images for validation of the segmentation algorithm.

A fractal pattern consisting of 6 branches sharing 2 branch points was generated (a). Linear interpolation method generated intermediate points for each branch (b). Intermediate 3D points within a branch were shifted by adding a small value randomly chosen using trignometric functions followed by spline interpolation and smoothening (see Methods) (c). A slice (of size 80×80 pixels) (d) from a reconstructed 3-dimensional noise image following a normal distribution as observed in the real EM data (d inset). 3D branch points converted into a 3D image of size 80×80×26 using a Gaussian function with added noise are shown (6 slices with slice number on left top) (e). Image segmentation algorithm with same parameters as in Fig 2 produced rendered image (f) and the corresponding skeleton image (g). For each run of the code, slight variations in branch lengths and branch angles between filaments were randomly produced for the same initial 6-branched fractal pattern (g-j). Branch points are highlighted in green and end points in black with branch lengths and angle between branches as indicated.

Figure S4. The localization of keratin and microtubules at the peridermal apical and sub-apical domains.

Confocal microscopy analysis of head peridermal cells showing localization of apical keratin (a), and sub-apical microtubules (b) at 48 hpf. Note that keratin follows the microridge pattern in parts but microtubules do not.

Images obtained from 48 hpf larvae. Scale bars represent 10 µm.

Figure S5. Formation of microridges during early embryogenesis.

Confocal microscopy analysis of developing microridges by phalloidin staining (a-i) and SEM (j-l) at the given stages. Note that at 9 hpf the actin punctae do not form projections in most cells except at the cell periphery (b,j). The microridges are already formed at the cell periphery at 18 hpf but not at the cell center and their length increases over time.

The scale bars are equivalent to 10 µm.

The images are representative for: Phalloidin stainings: a total of 10 animals from 2 sets; SEM: a total of 6 animals from 2 sets.

Figure S6. Quantification of microridge growth between 9 and 18 hpf.

Box plot showing the mean microridge length per cell for the time points 9, 12, 15, 18 hpf for two experimental sets of data. Each small circle represents the mean microridge length for a single cell and indicates individual data points used in the generation of the plot.

Figure S7. Qualitative analysis of Arp 2/3 complex inhibition by CK666.

Cells were qualitatively grouped into three classes based on their microridges. Figures a- i show representative images for each of the classes. (a,d,g) Low – broken down microridges or reduced number of punctae; (b,e,h) Medium – moderately broken down microridges or moderate reduction in punctae, and (c,f,i) High – intact microridges or intact punctae. The graph j shows experimental data from two sets depicting the relative proportion of the three classes of microridge effects at the given time-points. The graphs are based on the following data for a given treatment: (Numbers of animals in set 1, Number of cells in set 1; Numbers of animals in set 2, Number of cells in set 2). The numbers are: for 10 hpf - DMSO (7, 15; 5, 36); CK689 (7, 28; 5, 36); CK666 (7, 25; 5, 33). For 19 hpf - DMSO (6, 30; 6, 28); CK689 (6, 29; 6, 30); CK666 (6, 30; 6, 30). For 48 hpf - DMSO (5, 30; 5, 30); CK689 (5, 30; 5, 30); CK666 (5, 30; 5, 29). The representative phenotypic outcomes of these experiments are shown in Fig 5.

Scale bars are equal to 10 μm.

Figure S8. Keratin localization analysis during early development.

(a-c) Phalloidin staining at 9 (a), 12 (b), and 15 (c) hpf in red and corresponding keratin stainings in fire pseudocolour (d-f). Boxplots (g) showing keratin intensities for two separate experimental sets. There were 4-6 animals per set. The calibration bar for the keratin staining intensity in d is for d-f.

Scale bars correspond to 10 μm.

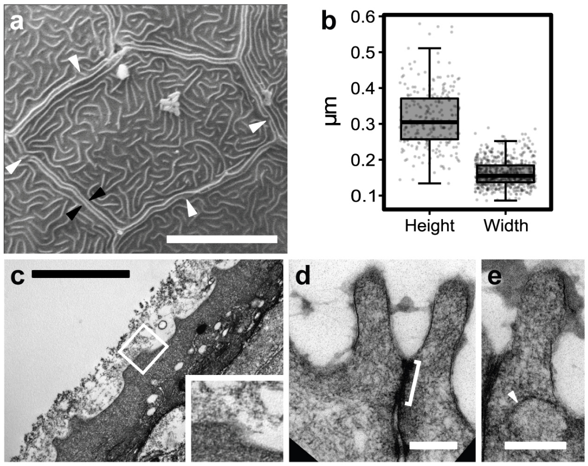

Figure S1

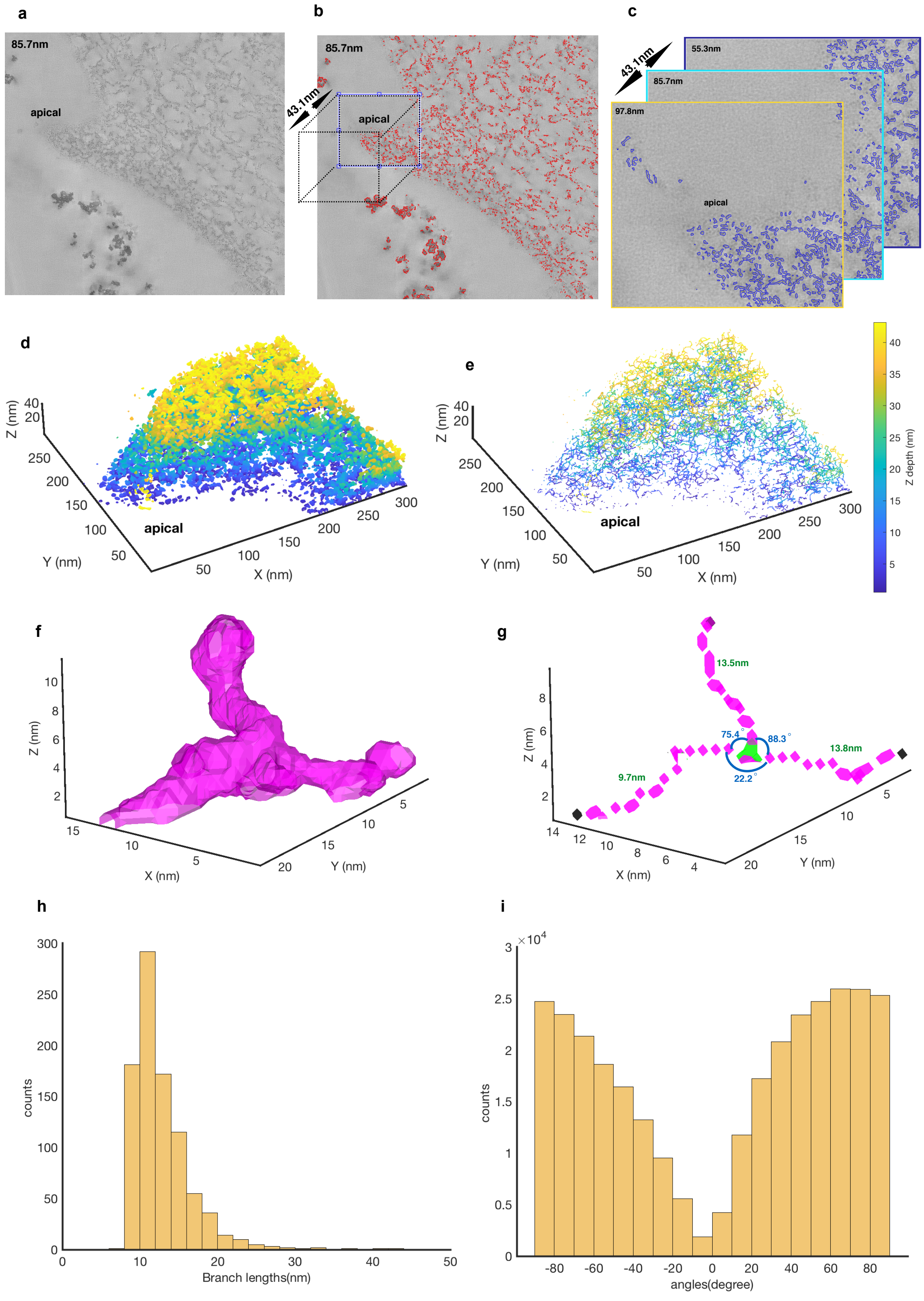
a
85.7nm
apical
b
85.7nm
43.1nm
apical
c
43.1nm
55.3nm
85.7nm
97.8nm
apical
d
Z (nm)
40
20
250
200
150
100
50
Y (nm)
apical
50
100
150
200
250
300
X (nm)
e
Z (nm)
40
20
250
200
150
100
50
Y (nm)
apical
50
100
150
200
250
300
X (nm)
Z depth (nm)
40
35
30
25
20
15
10
5
f
Z (nm)
10
8
6
4
2
15
10
5
X (nm)
5
10
15
20
Y (nm)
g
Z (nm)
8
6
4
2
13.5nm
75.4°
88.3°
22.2°
9.7nm
13.8nm
14
12
10
8
6
4
X (nm)
5
10
15
20
Y (nm)
h
counts
300
250
200
150
100
50
0
0
10
20
30
40
50
Branch lengths(nm)
i
×10^4
3
2.5
2
1.5
1
0.5
0
counts
-80
-60
-40
-20
0
20
40
60
80
angles(degree)

Figure S2

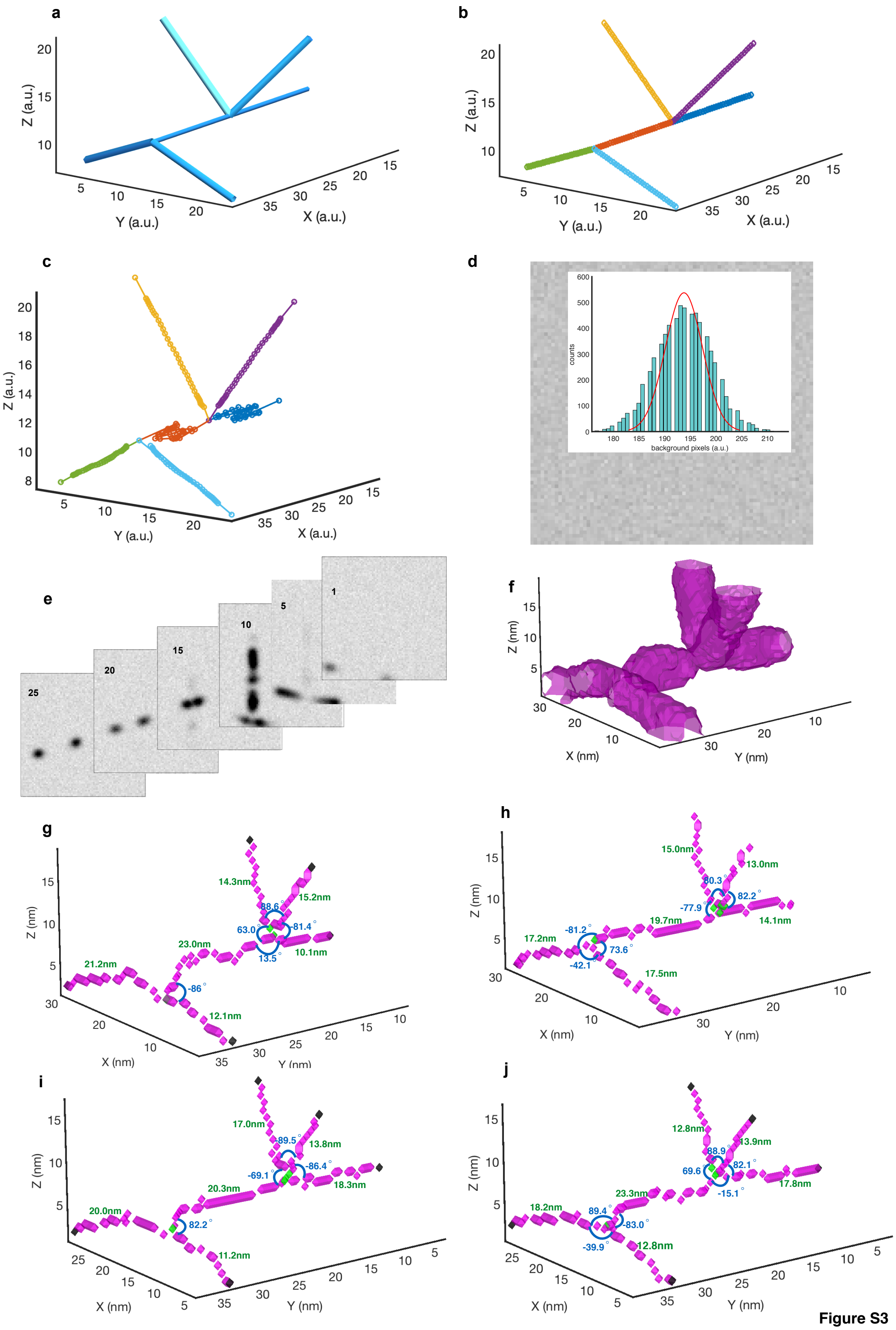
a
Z (a.u.)
Y (a.u.)
X (a.u.)
b
Z (a.u.)
Y (a.u.)
X (a.u.)
c
Z (a.u.)
Y (a.u.)
X (a.u.)
d
counts
background pixels (a.u.)
e
f
Z (nm)
X (nm)
Y (nm)
g
14.3nm
15.2nm
88.6
63.0
-81.4
13.5
10.1nm
23.0nm
21.2nm
-86
12.1nm
Z (nm)
X (nm)
h
15.0nm
13.0nm
80.3
-77.9
82.2
19.7nm
14.1nm
17.2nm
-81.2
73.6
-42.1
17.5nm
Z (nm)
X (nm)
Y (nm)
i
17.0nm
-89.5
13.8nm
-69.1
-86.4
18.3nm
20.3nm
20.0nm
82.2
11.2nm
Z (nm)
X (nm)
Y (nm)
j
12.8nm
88.9
13.9nm
69.6
82.1
-15.1
17.8nm
23.3nm
18.2nm
89.4
-83.0
-39.9
12.8nm
Z (nm)
X (nm)
Y (nm)

Figure S3

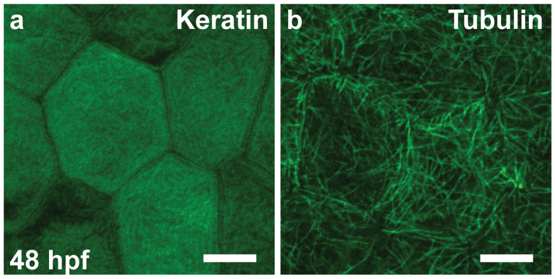

Figure S4

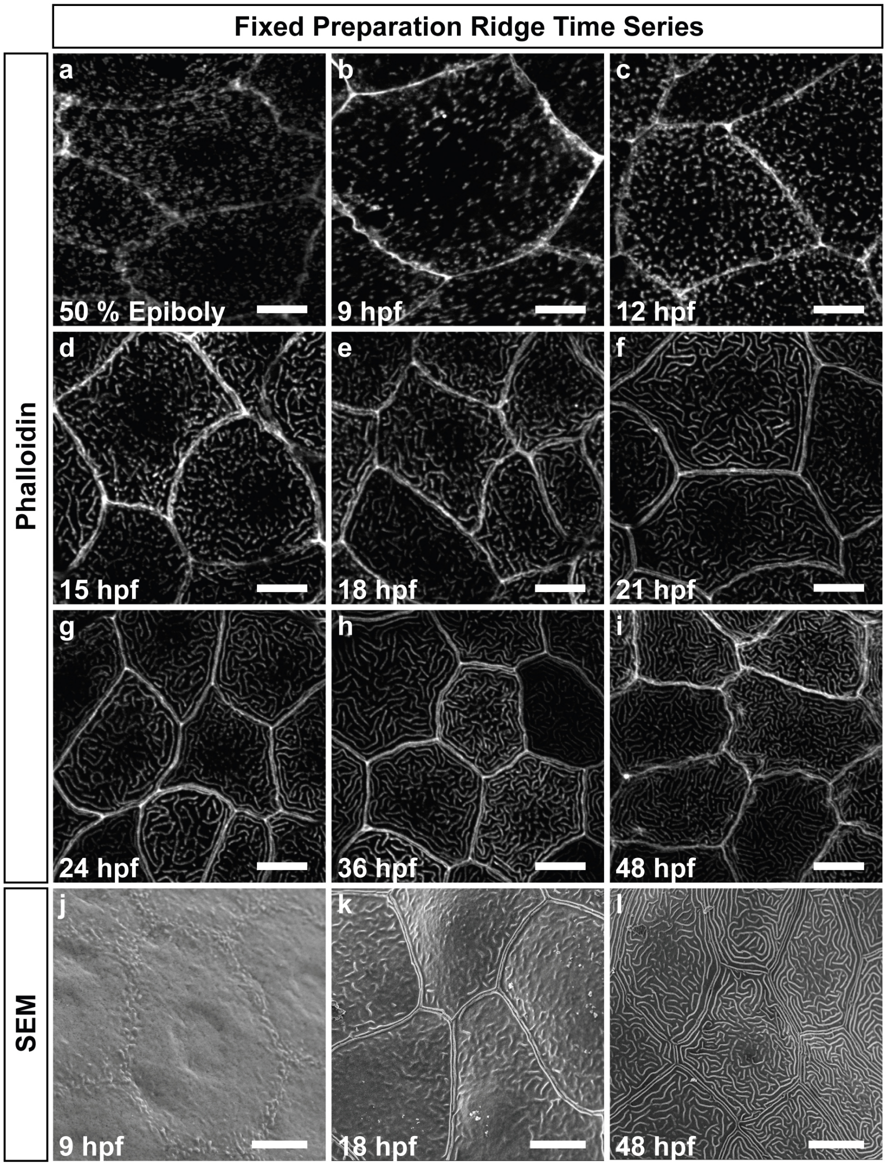
Fixed Preparation Ridge Time Series
Phalloidin
SEM
a
50 % Epiboly
b
9 hpf
c
12 hpf
d
15 hpf
e
18 hpf
f
21 hpf
g
24 hpf
h
36 hpf
i
48 hpf
j
9 hpf
k
18 hpf
l
48 hpf

Figure S5

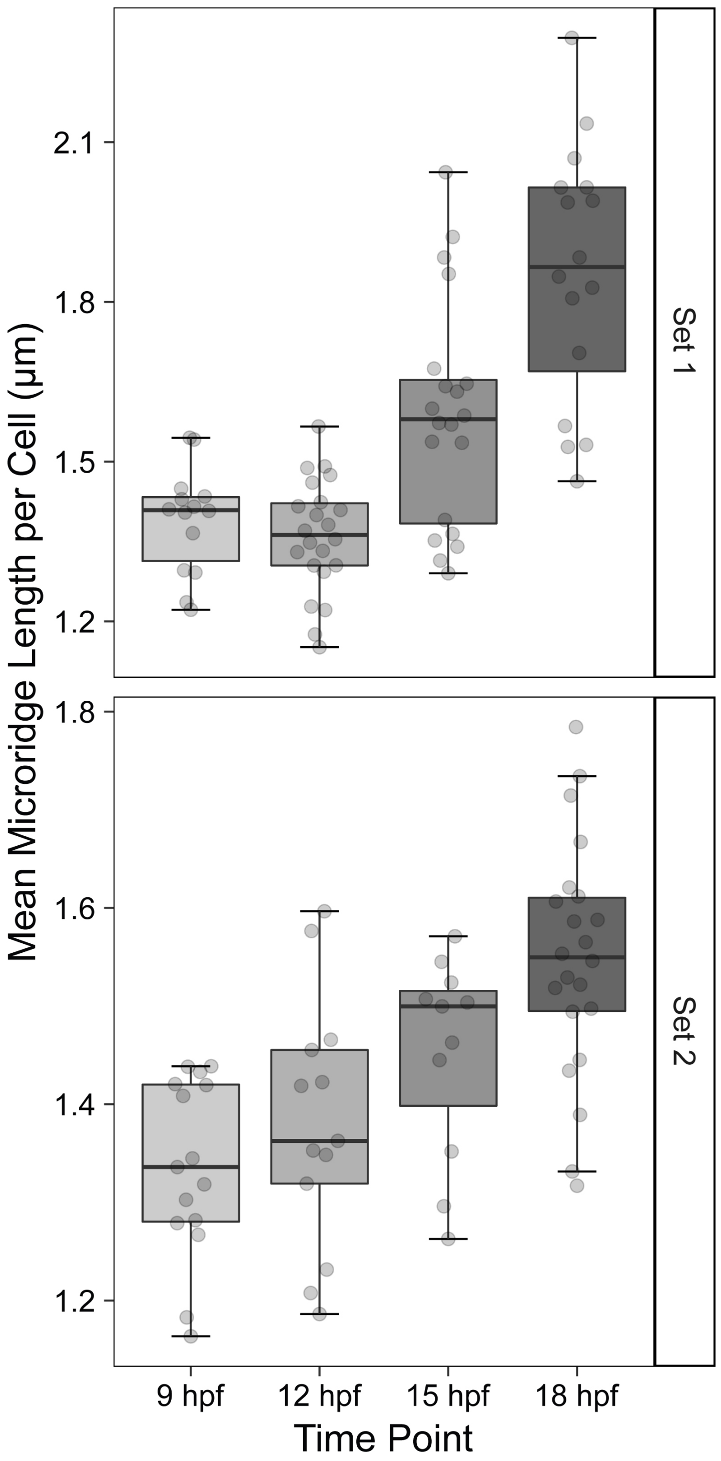
Mean Microridge Length per Cell (µm)
2.1
1.8
1.5
1.2
Set 1
1.8
1.6
1.4
1.2
Set 2
9 hpf
12 hpf
15 hpf
18 hpf
Time Point

Figure S6

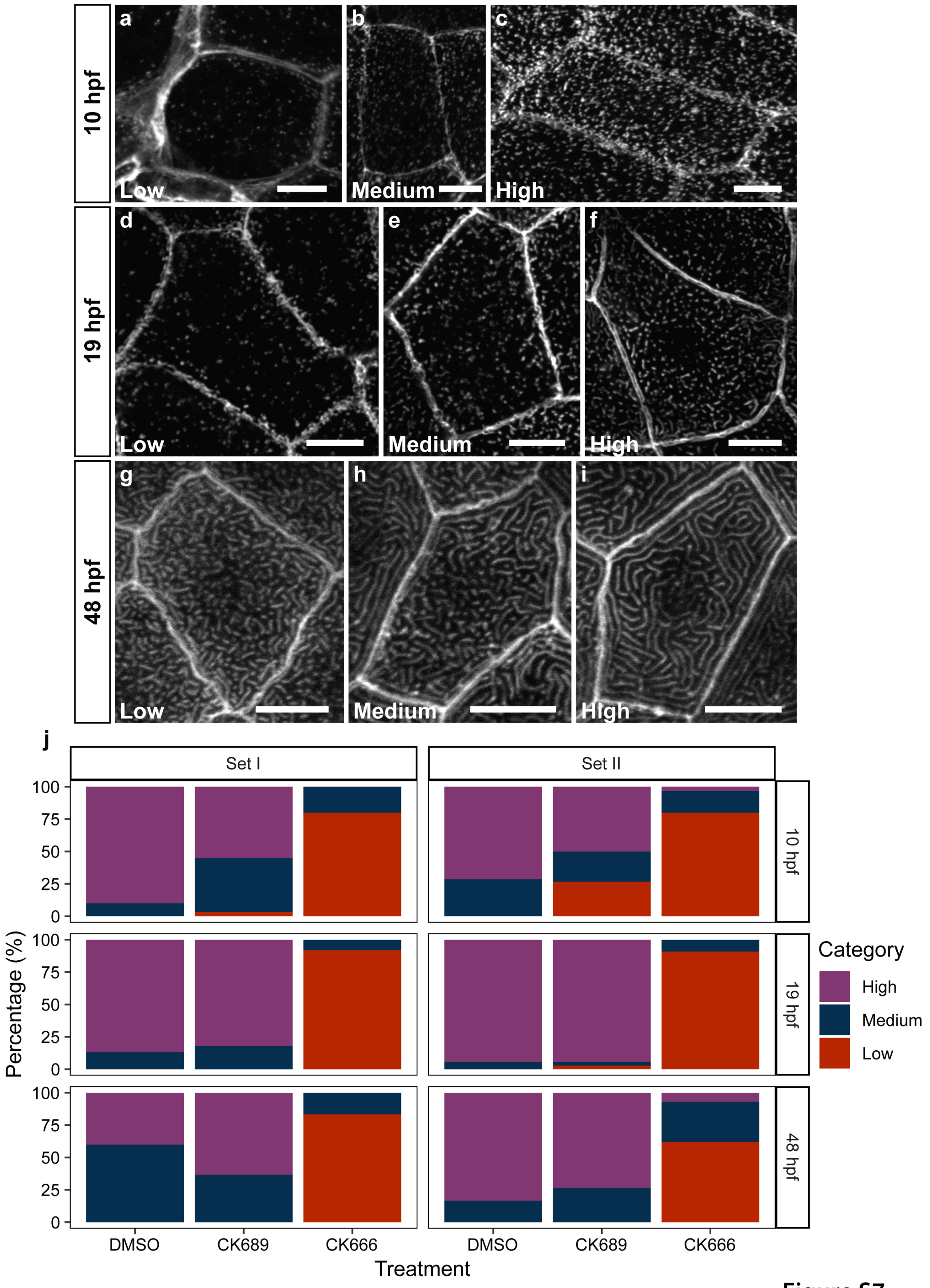
10 hpf
19 hpf
48 hpf
a
b
c
Low
Medium
High
d
e
f
Low
Medium
High
g
h
i
Low
Medium
High
j
Set I
Set II
100
75
50
25
0
Percentage (%)
10 hpf
19 hpf
48 hpf
Category
High
Medium
Low
DMSO
CK689
CK666
DMSO
CK689
CK666
Treatment

Figure S7

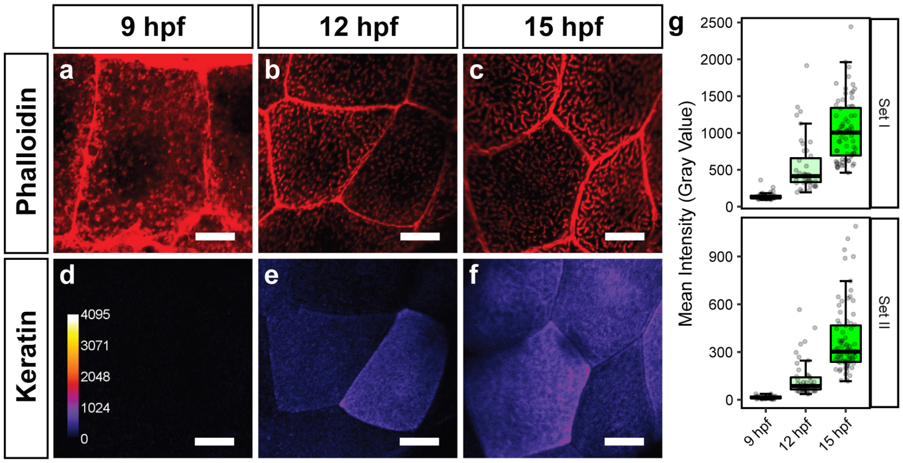
9 hpf
12 hpf
15 hpf
Phalloidin
Keratin
a
b
c
d
e
f
g
4095
3071
2048
1024
0
Mean Intensity (Gray Value)
Set I
Set II
2500
2000
1500
1000
500
0
900
600
300
0
9 hpf
12 hpf
15 hpf

Figure S8
